# Risk factors associated with deep perineal wound dehiscence after second‐degree perineal tears or episiotomy

**DOI:** 10.1111/aogs.70285

**Published:** 2026-06-18

**Authors:** Denise Golmann, Josefin Hedström, Elisabeth Epstein, Ida Bergman

**Affiliations:** ^1^ Department of Obstetrics and Gynecology Stockholm South General Hospital (Södersjukhuset) Stockholm Sweden; ^2^ Department of Clinical Science and Education, Stockholm South General Hospital Karolinska Institutet Stockholm Sweden

**Keywords:** antibiotic prophylaxis, episiotomy, pelvic floor dysfunction, perineal tear, vaginal delivery, wound dehiscence

## Abstract

**Introduction:**

Second‐degree perineal tears and episiotomies, involving the bulbocavernosus and/or superficial transverse perineal muscles, are common after vaginal birth and may lead to wound dehiscence, infection, and long‐term pelvic floor dysfunction. Despite this, risk factors for deep wound dehiscence involving perineal musculature remain insufficiently studied. This study aimed to identify risk factors for wound dehiscence in second‐degree tears and episiotomies to inform potential use of antibiotic prophylaxis.

**Material and Methods:**

A single‐center case–control study (1:2 ratio) was conducted including 105 cases with 2nd degree perineal tears or episiotomies complicated by deep wound dehiscence, diagnosed within two weeks postpartum and 210 controls with second‐degree tears or episiotomies without dehiscence. Exposure data were retrieved from medical records. Univariate analyses were performed on a priori selected exposure variables, using Mann–Whitney *U* and Fisher's exact tests. Variable selection used Lasso regression with leave‐one‐out cross‐validation. Multivariate logistic regression generated adjusted odds ratios (aOR). Statistical significance was set at *p* < 0.05.

**Results:**

Women with wound dehiscence were more often vaginal primiparas (87% vs 69%, *p* < 0.001) and had higher rates of episiotomy (33% vs 7%, *p* < 0.001). Intrapartum antibiotics were administered less frequently among cases (7% vs 20%, *p* = 0.002). Cases had longer second stage of labor, more active pushing, and greater postpartum bleeding. After multivariate adjustment, only episiotomy (aOR 4.40, 95% CI 2.10–9.64) and intrapartum antibiotic administration (aOR 0.21, 95% CI 0.07–0.50) remained significantly associated with wound dehiscence.

**Conclusions:**

Episiotomy substantially increased the risk of deep perineal wound dehiscence compared with spontaneous second‐degree perineal tears, while intrapartum antibiotics were protective. These findings support selective episiotomy use and suggest that antibiotic prophylaxis may reduce this risk in women with second‐degree perineal tears or episiotomies.

## Graphical abstract

Episiotomy and intrapartum antibiotic administration were the two independent predictors of deep perineal wound dehiscence in second‐degree tears and episiotomies. Episiotomy increased dehiscence risk fourfold (aOR 4.40), while intrapartum antibiotics were protective (aOR 0.21). Findings support selective episiotomy use and suggest antibiotic prophylaxis may reduce wound breakdown.

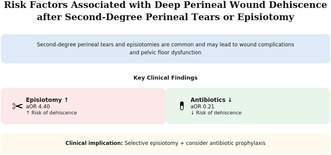

This article links with AOGS_105_7 issue.

**Abbreviations**
aOR: adjusted odds ratio
BMI: body mass index
CI: confidence interval
ICD: international classification of diseases
OR: odds ratio
VE: vacuum extraction

**Key message**

Our study indicates that episiotomy is a major risk factor for deep wound dehiscence after second‐degree perineal tears. Intrapartum antibiotic administration substantially reduces this risk.

## 1 INTRODUCTION

After vaginal delivery most women sustain perineal tears involving musculature, classified as second‐, third‐ or fourth‐degree perineal tears.^1^ Extensive research has been conducted on how to prevent, diagnose and treat the more severe third and fourth‐degree tears. A second‐degree perineal tear involves the bulbocavernous muscle and/or the superficial transverse perineal muscle, but not the anal sphincter muscles. The bulbocavernous muscle is a thin striated muscle that is important for sexual function as it contributes to the clitoral function and it also acts as a closing muscle for the vaginal orifice.^2^, ^3^ The transverse perineal muscle is also a striated muscle that is crucial for the support of the anal canal and the posterior vaginal wall as it anchors the perineal body and surrounding soft tissue to the pelvic bone.^3^, ^4^, ^5^ Poorly healed second‐degree perineal tears or episiotomies can lead to sexual dysfunction and pelvic floor‐related problems with major impact on the quality of life.^6^, ^7^ They can be treated later in life with reconstructive pelvic floor surgery, which comes with risks of long‐term complications such as pain and recurrence.^8^

Reasons for poorly healed perineal tears include poor primary suturing technique, wound infection, and wound dehiscence (meaning that the perineal wound breaks down partially or totally after the primary suturing). A perineal wound dehiscence is often accompanied by an infection, but it is difficult to establish cause and effect. It is common knowledge that infections can lead to surgical wound breakdown in general. As the perineal body is situated between the anal canal and the vagina, two areas with high bacterial load, it is plausible to assume that when the protective skin barrier is broken, the risk of a secondary infection is high.

As not much research has focused on second‐degree perineal tears and episiotomies, there are knowledge gaps regarding the incidence and risk factors of wound dehiscence of these tears. Another complicating factor is that most studies on perineal wound dehiscence make no distinction between superficial skin dehiscence and deeper dehiscence involving musculature.

To the best of our knowledge, no previous study has assessed risk factors for wound dehiscence involving perineal musculature in women with second‐degree perineal tear. The aim of this study is to address this knowledge gap and identify possible groups of women that could benefit from antibiotic prophylaxis.

## 2 MATERIAL AND METHODS

### 2.1 Study design

Case–control study 1:2.

### 2.2 Study population^9^

In total 315 women were included: 105 cases and 210 controls.

The study included women with vaginal deliveries complicated by a second‐degree perineal tear or an episiotomy at a tertiary hospital in Stockholm, Sweden with about 6000 deliveries annually, during the period of May 17, 2017–December 31, 2022.

Cases are defined as women with second‐degree perineal tears or episiotomies complicated by wound dehiscence diagnosed within two weeks postpartum. Wound dehiscence is defined as a separation of perineal muscles involving at least the entire bulbocavernous muscle. Women with third and fourth‐degree tears were excluded. Both cases and controls were offered a routine perineal assessment before discharge from the maternity ward. The cases were identified during clinical assessment in a pilot study and an ongoing randomized controlled trial (RCT) comparing treatment strategies for dehisced perineal tears. Women were referred to the study investigators because of dehisced perineal tears, and those with rupture involving at least the entire bulbocavernosus muscle were eligible for inclusion. The patients were referred after a median of 5 days postpartum (IQR 4–8 days).

Controls were women with vaginal deliveries complicated by second‐degree tears or episiotomies, at the same hospital as the cases. Controls were selected in a 2:1 ratio and were matched by date of delivery and temporal proximity to the case. Controls were identified through medical record review using International Classification of Diseases (ICD)‐10 codes. Women were classified as controls if they had a documented second‐degree tear or an episiotomy, and no documented wound dehiscence in the medical record. Controls were not invited to a planned study‐specific follow‐up visit; thus, control classification relied on routine clinical follow‐up and documented representation to care.

The controls were identified if they had a second‐degree perineal tear (O70.1), episiotomy (TMA00) and having no wound dehiscence (090.1).

### 2.3 A priori decided exposure variables

A priori decided exposure variables included antepartum, intrapartum, and postpartum factors. Antepartum exposures were age (years), body mass index (BMI; WHO classes), parity (vaginal nulliparous vs multiparous), smoking during pregnancy (yes/no). Intrapartum exposures were onset of labor (spontaneous or induction), episiotomy (yes/no), instrumental delivery (yes/no), location of perineal suturing (operating theater or delivery ward), professional performing perineal suturing (obstetrician or midwife), duration of second stage of labor (minutes), duration of active pushing (minutes), time from delivery until perineal suturing (minutes), epidural anesthesia (yes/no), meconium‐stained fluid (yes/no), intrapartum antibiotics (yes/no), and head circumference (cm). Postpartum exposures included postpartum bleeding (mL) and postpartum bleeding ≥1000 mL. Exposure variables were retrieved from the medical records.

### 2.4 Statistical analyses

The collected patient data were analyzed based on a priori decided exposure variables. Data on baseline characteristics were analyzed with Stata version 17 (StataCorp LLC, College Station, TX, USA) presented as frequencies and percentage distributions for categorical variables and as medians with range for continuous variables. Univariate comparisons of baseline characteristics between groups were performed with the Mann–Whitney U test for continuous variables and Fisher's exact test for categorical variables. Statistical significance was set to *p* < 0.05.

Because a relatively large number of candidate exposure variables were considered in relation to the number of outcome events, LASSO regression was used as a variable selection tool to reduce the risk of overfitting when evaluating multiple correlated obstetric variables. LASSO regression with leave‐one‐out cross‐validation was applied to identify variables most strongly associated with the outcome. Variables suggested by the penalized model were subsequently reviewed based on clinical relevance before inclusion in the final multivariable logistic regression model. For example, postpartum hemorrhage (mL) was not included in the final model although it was statistically significant, as we considered the variable postpartum hemorrhage ≥1000 mL to be more clinically relevant, given its potential to influence the risk of infection and wound dehiscence through anemia. Crude associations were explored using univariable logistic regression, and the final adjusted estimates were derived from multivariable logistic regression.

## 3 RESULTS

Baseline characteristics (presented in Table 1). The cohort had a median age of 32 years and a median BMI of 23; the majority were non‐smokers. A significant difference between groups was seen in vaginal primiparity, where 87% of women with wound dehiscence were vaginal primiparas while only 69% of women with no wound dehiscence (*p* < 0.001). Further, episiotomy, duration of the second stage of labor, time of active pushing, and postpartum hemorrhage (mL) showed a significant difference between cases and controls. Intrapartum antibiotics were administered less frequently among cases than controls (7% vs 20%, *p* = 0.002).

**TABLE 1:** Baseline characteristics of the study population.

Characteristic	Wound dehiscence (*N* = 105)	No wound dehiscence (*N* = 210)	*p*‐value	Missing (%)
Age (y)	32 (18–39)	32 (21–43)	0.68	0
BMI (kg/m^2^)	23 (18–33)	23 (17–42)	0.82	1.9
Smoking	3 (3)	8 (4)	0.68	0.3
*Parity*				
Vaginal primipara	91 (87)	144 (69)	<0.001	0
Previous caesarean section	2 (2)	12 (6)	0.15	0
Induction	25 (24)	54 (26)	0.78	0
Episiotomy	35 (33)	15 (7)	<0.001	0
Instrumental vaginal delivery (VE)	27 (26)	22 (11)	<0.001	0
Active pushing (min)	36 (5–262)	25 (3–92)	<0.001	2.9
Second stage of labor (min)	142 (5–463)	82 (3–373)	<0.001	4.4
Intrapartum antibiotics	7 (7)	42 (20)	0.002	0
Head circumference (cm)	35 (31–38)	35 (31–39)	0.57	0.06
Birthweight (g)	3640 (2660–3920)	3605 (2310–4710)	0.23	0
Epidural	71 (68)	134 (64)	0.53	0
Meconium‐stained amniotic fluid	26 (25)	42 (20)	0.38	0.3
Postpartum hemorrhage (mL)	550 (150–3200)	400 (75–2650)	0.005	0
Postpartum hemorrhage ≥1000 mL	17 (16)	20 (10)	0.096	0
Time from delivery to primary suturing (min)	45 (3–601)	33 (4–592)	0.002	0.09
*Clinician performing primary suturing*				
Attending ob/gyn	59 (56)	75 (36)	<0.001	0
Midwife	46 (44)	135 (64)		0
*Location of perineal suturing*				
Operation theater	16 (15)	14 (7)	0.023	0
Delivery room	89 (85)	196 (93)		0

*Note*: Postpartum bleeding includes all measured bleeding during birth and postpartum care. Data are median (range) or *n* (%) unless otherwise specified.

Abbreviations: BMI, body mass index; VE, vacuum extraction.

Variables retained in the final multivariable model following statistical variable selection and clinical assessment were episiotomy, vaginal primiparity, intrapartum antibiotics, location of primary suturing, instrumental delivery, postpartum hemorrhage ≥1000 mL, second stage of labor, and active pushing.

Potential risk factors for wound dehiscence in second‐degree perineal tears are presented as crude odds ratios (OR) and confidence intervals (CI) and as adjusted odds ratios (aOR) with CI presented in Table 2. Multivariate adjustment was performed using all exposure shown in Table 2. After adjusting for confounding factors, only two variables remained statistically significant (Table 2). The risk of wound dehiscence increased with an aOR of 4.40 (95% CI 2.10–9.64) in women who sustained an episiotomy during vaginal delivery compared to women with a second‐degree perineal tear. Administration of intrapartum antibiotics during vaginal delivery decreased the risk of wound dehiscence with an aOR of 0.21 (95% CI 0.07–0.50).

**TABLE 2:** Risk factors for perineal wound dehiscence.

Characteristic	Category	*N* (Crude)	OR	95% CI	*p*‐value	*N* (Adjusted)	aOR	95% CI	*p*‐value
Episiotomy	No	265	–	–	–	243	–	–	–
	Yes	50	6.50	3.41–12.9	<0.001	50	4.40	2.10–9.64	<0.001
Vaginal primipara	No	80	–	–	–	63	–	–	–
	Yes	235	2.98	1.62–5.81	<0.001	230	1.77	0.79–4.15	0.2
Intrapartum antibiotics	No	266	–	–	–	246	–	–	–
	Yes	49	0.29	0.11–0.62	0.003	47	0.21	0.07–0.50	0.001
Location of perineal suturing	Obstetric ward	285	–	–	–	265	–	–	–
	Operation theater	30	2.52	1.18–5.45	0.017	28	2.00	0.80–5.08	0.14
Instrumental delivery (VE)	No	266	–	–	–	246	–	–	–
	Yes	49	2.96	1.59–5.55	<0.001	47	1.21	0.54–2.67	0.6
Postpartum hemorrhage ≥1000 mL	No	278	–	–	–	257	–	–	–
	Yes	37	1.84	0.91–3.67	0.086	36	1.12	0.48–2.51	0.8
Second stage of labor (min)	–	301	1.01	1.00–1.01	<0.001	293	1.00	1.00–1.01	0.2
Active pushing (min)	–	306	1.03	1.02–1.04	<0.001	293	1.01	1.00–1.03	0.12

Abbreviations: aOR, adjusted odds ratio; CI, confidence interval; OR, odds ratio; VE, vacuum extraction.

## 4 DISCUSSION

Our study indicates that episiotomy was associated with an approximately fourfold increased occurrence of deep wound dehiscence involving perineal musculature in women with second‐degree tears and/or episiotomy, whereas intrapartum antibiotic administration was associated with a substantially lower occurrence.

The apparent protective association between intrapartum antibiotics in our study is consistent with recent evidence from randomized trials. The REPAIR study, a single‐center double‐blind placebo‐controlled randomized trial in women with episiotomy or second‐degree tears, reported a reduction in clinically relevant wound complications after prophylactic antibiotic administration, and importantly this effect was also seen among women without operative vaginal delivery^10^ Similarly, the ANODE trial demonstrated reduced wound complications after operative vaginal delivery with prophylactic antibiotics.^11^ Although the ANODE population was defined by operative delivery, most women had episiotomy or second‐degree tears. Together, these trials support the hypothesis that the perineal trauma itself, rather than instrumentation alone, may be the key substrate for postpartum wound complications.

Possible pathophysiological mechanisms underlying the protective association between antibiotics and reduced wound dehiscence include reduction of bacterial colonization and secondary infection in traumatized perineal tissue, thereby limiting local inflammation, tissue edema, and impaired wound healing.

Systematic reviews have also demonstrated wide variation in reported incidences of infection and wound dehiscence after childbirth‐related perineal trauma and after obstetric anal sphincter injury repair, largely because of differences in definitions, follow‐up procedures, and outcome ascertainment. Jones et al. reported wound infection rates ranging from 0.1% to 23.6% and dehiscence rates from 0.21% to 24.6% across childbirth‐related perineal trauma populations.^12^ Okeahialam et al. reported overall incidences of 4.4% for wound infection and 6.9% for wound dehiscence after primary OASI repair, with wide prediction intervals.^13^ More recently, Perslev et al. showed that studies with planned clinical follow‐up 1–3 weeks postpartum reported higher prevalences of complications after second‐degree tears and episiotomy than studies without such evaluations.^14^

This observation is important when interpreting our findings. Cases in the present study were identified through clinical assessment within two weeks postpartum, whereas controls were classified based on routine care and absence of documented dehiscence. It is therefore possible that some wound complications in the control group were not detected. Such underascertainment would tend to bias estimates toward the null, but surveillance bias may also have affected the apparent association with episiotomy if women with episiotomy were more likely to receive information about wound complications or to represent to care.

Evidence suggests that episiotomy should be used selectively rather than routinely in unselected populations.^15^ At the same time, recent evidence such as the EVA trial suggests that lateral episiotomy may reduce obstetric anal sphincter injury in nulliparous women undergoing vacuum extraction.^16^ This broader clinical context highlights that even where episiotomy may be beneficial in selected circumstances, prevention of downstream wound complications remains important.

The incidence of episiotomy and recommendations regarding when to perform the procedure vary considerably both between and within countries.^17^, ^18^ For example, the incidence of episiotomy in non‐instrumental vaginal deliveries has been reported to be 3.7% in Denmark and 75% in Cyprus.^19^ These discrepancies suggest that further efforts could be made to reduce unnecessary episiotomies and thereby decrease the occurrence of more severe types of perineal wound dehiscence involving musculature. The results of our study are consistent with previous research and suggest that antibiotic prophylaxis should be considered for women undergoing episiotomy, as episiotomy appears to be a strong risk factor for wound infection and dehiscence. If a selective approach to episiotomy is applied, the benefits of reducing both short and long‐term perineal wound complications^6^ are likely to outweigh the risks associated with antibiotic prophylaxis. If deep perineal wound dehiscence is prevented, the need for later reconstructive procedures such as perineorrhaphy may potentially be reduced.^6^, ^7^ Whether women undergoing episiotomy would benefit from more extensive antibiotic treatment than single‐dose prophylaxis remains unclear and should be evaluated in future prospective studies.

In a prospective cohort study from Denmark, obesity was found to be the strongest risk factor for wound dehiscence in any degree of perineal tear.^20^ In our study, BMI did not differ between cases and controls, but our cohort was not powered to assess obesity as an isolated exposure.

A limitation in our study is that detailed subclassification of second‐degree tears was not available. Recent publications have proposed more detailed subclassifications of second‐degree tears,^21^ which will be valuable in future work and may improve risk stratification.

Another limitation of the present study is that the indication for intrapartum antibiotic treatment was not sufficiently standardized in the retrospective data source to allow meaningful subgroup analyses according to indication. Furthermore, information regarding postpartum antibiotic administration was not available in the retrospective data source. Consequently, we could not assess whether postpartum antibiotic treatment may have influenced the risk of wound complications or modified the observed associations.

The case–control design allowed efficient identification of women with a relatively uncommon outcome and enabled evaluation of several candidate risk factors. By selecting controls with the same degree of tear, from the same hospital and day of delivery, we attempted to increase similarity between groups regarding contextual factors such as workload and local practice.

As this was a single‐center study it limits the external validity and generalizability of the results. A multicenter study would allow broader generalization of the results and strengthen the external validity. When interpreting the results, it is important to consider that the sample size may have been insufficient for some of the studied exposure variables.

To the best of our knowledge, this is the first study specifically evaluating risk factors for deep perineal wound dehiscence involving disruption of the perineal musculature after second‐degree tears or episiotomy. Previous studies, including the ANODE and REPAIR trials, have demonstrated reduced postpartum wound complications following antibiotic prophylaxis but did not provide detailed information regarding the anatomical depth of wound dehiscence or whether the disruption involved the perineal musculature. Consequently, the present study contributes additional information regarding risk factors for the more severe spectrum of perineal wound dehiscence, which may be of particular clinical relevance as deep dehiscence involving musculature is likely associated with greater morbidity and may require reconstructive treatment.

To facilitate research on risk factors for perineal wound complications following vaginal delivery we recommend that core outcome sets are established with clear‐cut definitions of perineal wound dehiscence, making distinction between superficial and deep dehiscence.

## 5 CONCLUSION

Intrapartum antibiotic administration was associated with a lower occurrence of deep wound dehiscence involving perineal musculature among women with second‐degree tears or episiotomy, while episiotomy was associated with increased occurrence. These findings support selective use of episiotomy and suggest that antibiotic prophylaxis may be relevant in preventing perineal wound complications.

## AUTHOR CONTRIBUTIONS

DG, EE, and IB conceived the study and agreed on the study design. DG, IB, and EE obtained funding. The statistical analyses were performed in collaboration with a statistician at the KI Biostatistics Core Facility. DG drafted the manuscript. All authors contributed to data interpretation, critically revised the manuscript, and approved the final version. IB acted as guarantor.

## FUNDING INFORMATION

The study was funded by Swedish governmental funding of clinical research (ALF) and by the Swedish Research Council (2020‐00711). The funders had no role in study design, data collection, analysis, interpretation, manuscript preparation, or the decision to submit the article for publication.

## CONFLICT OF INTEREST STATEMENT

The authors declare that no conflicts of interest exist.

## ETHICS STATEMENT

The study was approved by the Swedish Ethical Review Authority (2022‐00354‐01, approved on 28 March 2022).

## Data Availability

The data that support the findings of this study are available on request from the corresponding author. The data are not publicly available due to privacy or ethical restrictions.
